# Fucoidan Structure and Its Impact on Glucose Metabolism: Implications for Diabetes and Cancer Therapy

**DOI:** 10.3390/md19010030

**Published:** 2021-01-11

**Authors:** Blessing Mabate, Chantal Désirée Daub, Samkelo Malgas, Adrienne Lesley Edkins, Brett Ivan Pletschke

**Affiliations:** 1 Enzyme Science Programme (ESP), Department of Biochemistry and Microbiology, Rhodes University, Makhanda 6140, South Africa; bmabate@gmail.com (B.M.); chantaldaub97@gmail.com (C.D.D.); samkelo.malgas@up.ac.za (S.M.); 2 Biomedical Biotechnology Research Unit, Department of Biochemistry and Microbiology, Rhodes University, Makhanda 6140, South Africa; a.edkins@ru.ac.za

**Keywords:** fucoidan, natural bioproducts, antidiabetic, anticancer, glucose metabolism

## Abstract

Fucoidans are complex polysaccharides derived from brown seaweeds which consist of considerable proportions of L-fucose and other monosaccharides, and sulphated ester residues. The search for novel and natural bioproduct drugs (due to toxicity issues associated with chemotherapeutics) has led to the extensive study of fucoidan due to reports of it having several bioactive characteristics. Among other fucoidan bioactivities, antidiabetic and anticancer properties have received the most research attention in the past decade. However, the elucidation of the fucoidan structure and its biological activity is still vague. In addition, research has suggested that there is a link between diabetes and cancer; however, limited data exist where dual chemotherapeutic efforts are elucidated. This review provides an overview of glucose metabolism, which is the central process involved in the progression of both diseases. We also highlight potential therapeutic targets and show the relevance of fucoidan and its derivatives as a candidate for both cancer and diabetes therapy.

## 1. Introduction

Fucoidan is a complex sulphated polysaccharide found mainly in various species of brown seaweeds [1,2]. To date, fucoidans from various brown seaweeds have been partially characterised and studied for their potential bioactivities [3,4]. The bioactivity of fucoidans is linked to their degree of sulphation, monosaccharide composition, and molecular weight. Fucoidan composition is varied in different species, although extraction methods and seaweed harvest time may also influence the structural composition of the bio-compound extracted from the same species [5]. Despite the increased interest in brown seaweeds as sources of fucoidan, there are some gaps linking improved yield, composition and structure to bioactivity [2,6]. Furthermore, to date, there are only a few commercially available fucoidan extracts, limited to those isolated from *Fucus vesiculosus, Macrocystis pyrifera* and *Undaria pinnatifida* [2], yet vast biodiversity of unexplored seaweeds exists. Fucoidan’s numerous bioactive properties have been noted in both in vivo and in vitro studies [7]. These bioactivities include: anti-oxidant, anti-coagulant, anti-thrombotic, anti-inflammatory, anti-viral, anti-lipidemic, anti-metastatic, anti-diabetic and anti-cancer activities [7]. Importantly, fucoidan has been reported to have anticancer properties in both in vivo and in vitro studies. The mechanisms of action of exactly how fucoidan inhibits the metabolic pathways of tumour cells have been shown and are relatively well understood [8,9]. Although several studies have focused on pathways that destroy or slow down cancer progression, very few have focused on glucose metabolism pathophysiology, which is essential for the survival of cancer cells and a determining factor in diabetes progression.

Furthermore, fucoidan has been implicated as a plausible antidiabetic agent as some fucoidans have inhibited the primary starch digesting enzymes; amylase and glucosidase, directly linked to postprandial hyperglycaemia [5,10]. Nevertheless, limited studies have investigated the potential therapeutic effects of fucoidan on the several possible control points of glucose metabolism. Notably, diabetes and cancer progression are linked with shared factors which involve glucose metabolism pathophysiology [11]. Therefore, this review provides an overview of glucose metabolism, highlighting the possible therapeutic targets for fucoidan, as it has demonstrated dual biological activity against cancer and diabetes.

## 2. Fucoidan Structure

### 2.1. Fucoidan Backbone & Monosaccharide Composition

Fucoidans vary in their structure and composition but are primarily composed of a pentose sugar backbone comprised of fucose residues that are linked by α-(1–3), α-(1–3)-α-(1–4) or α-(1–3)-α-(1–2) linkages [1]. Fucoidans are heterogeneous in their monosaccharide composition with different monosaccharides occurring in different fucoidans extracted from various species and their proportions varying depending on the extraction process employed. The numerous neutral monosaccharides reported constituting fucoidans include glucose, galactose, xylose, mannose and rhamnose [6,12]. Furthermore, fucoidan may contain acetate and uronic acids, including glucuronic acid and galacturonic acid [13,14]. The monosaccharide units may substitute molecular entities on the fucoidan structure or may represent contamination of the fucoidan extracts with other polysaccharides [12].

### 2.2. Sulphate Content and Position

The sulphate content and overall degree of sulphation in fucoidans vary significantly between species. Variations in fucoidan sulphate content as a function of harvests over different seasons have also been reported numerous times in literature. For instance, varying sulphate content was reported among three species, namely, *F. serratus*, *F. vesiculosus* and *A. nodosum,* and within species harvested in different seasons [15]. The study further noted that the fucose and sulphate contents varied proportionally to each other and were inversely proportional to the total fucoidan content. A study on the galactofucans from *Saccharina longicruris* reported a 1.6% increase in sulphate content between March and November 2005, while the sulphate content decreased by 7.2% between November 2005 and June 2006 [15]. The sulphate group positioning is also one of the main qualities of fucoidan that determines its structural and functional capabilities [16]. It has been established that single and double sulphate group substitutions occur at the C-2 or C-4 positions of furanose residues constituting two types of fucoidan chain structures (the (1→3)-α-L-fucopyranose residues and α-L-fucopyranose residues linked by (1→3) and (1→4) glycosidic bonds [16]. Moreover, sulphate substitutions in fucoidans also occur on the C-2 and C-3 positions of other monosaccharide residues [17]. The methodologies employed in the determination of sulphate content within fucoidans include infrared spectroscopy (IR), de-sulphation, the stability of sulphate esters to alkali and methylation analysis [7]. Nuclear magnetic resonance (NMR) and mass spectroscopy can also be used to analyse the presence and positioning of sulphate residues on fucoidan [7]. 

### 2.3. Molecular Weight

The molecular weight of fucoidans has been one of the many factors affecting their functional properties. Fucoidan size varies from 10 kDa to about 10,000 kDa depending on the fucoidan source with the average size being approximately 20 kDa. [17]. The considerable variation in the size of fucoidans has led to the categorisation of fucoidans; low molecular weight fucoidan (LMWF) when <10 kDa in size, medium molecular weight fucoidan (MMWF) if between 10 and 10,000 kDa in size and high molecular weight fucoidan (HMWF) when >10,000 kDa in size [13]. Native fucoidan is known to have a high molecular weight, which results in low cell membrane permeability, low bioavailability, efficiency, and potential clinical efficacy [18]. There is a lot of variation in fucoidan characteristics (Table 1) due to various species, extraction protocols, and techniques used to determine traits. For example, determining the fucose content varies when less specific methods like the phenol sulphuric acid, which measures total carbohydrate content, are used—compared to more specific methods, including enzymatic assays and HPLC. These discrepancies make comparison very difficult. 

Depolymerisation of high-molecular-weight fucoidan to synthesise polysaccharide oligomeric components has recently gained attention as it may solve high molecular weight problems of native fucoidan [18]. For instance, native fucoidan (5–100 kDa) from *Undaria pinnatifida* had minimal anti-tumour activity compared to its depolymerised counterpart (490 kDa) [17]. Furthermore, a fucoidan fraction with a molecular weight range of 50–100,000 kDa was reported to be a potential anticoagulant, whilst fractions >850 kDa lacked activity [17]. Similarly, low molecular weight fucoidans (from 4.58 to 6.5 kDa) displayed high anticoagulant and antioxidant activity, which was explained by their greater solubility and bioavailability [18]. Therefore, it is evident that molecular weight is a vital factor in the bioactivity and bioavailability of fucoidan. Hence depolymerisation processes that will not cleave functional fucoidan side chains are necessary.

## 3. Overview of Glucose Metabolism

Glucose is the most prominent energy substrate for most organisms, including humans. The amount of glucose available in the bloodstream is mainly dependent on several processes, including, carbohydrate digestion, hormonal regulation, glucose transport mechanisms and glycolysis [24]. 

### 3.1. Carbohydrate Digestion and Absorption

**Figure 1:**
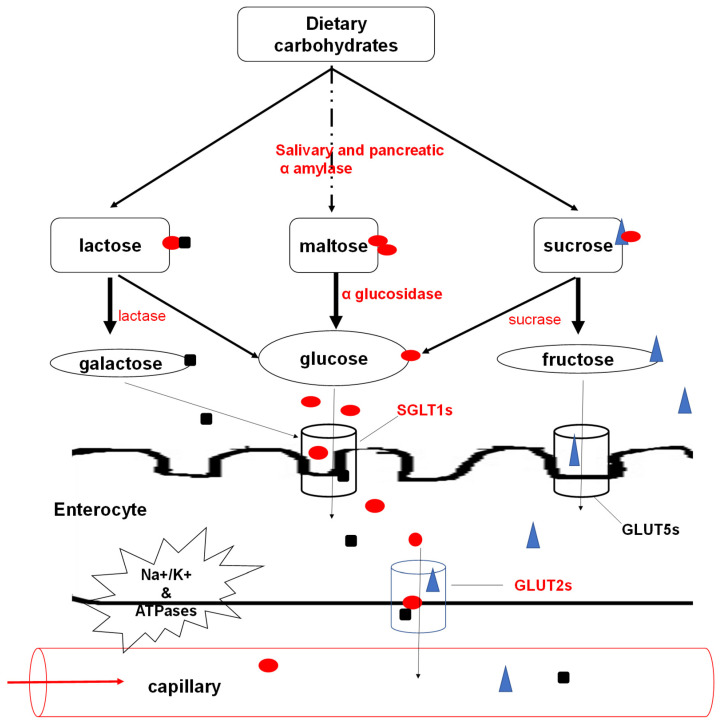
Carbohydrate digestion, absorption and assimilation. For simplicity, the monosaccharides are depicted in different shapes; squares represent galactose, circles glucose and triangles fructose. The steps of carbohydrate digestion are shown with corresponding hydrolytic enzymes (shown in red). The monosaccharide transporter molecules are shown as cylindrical shapes. The highlighted steps can be therapeutic targets for fucoidan. Adapted from [27].

The primary glucose supply for the human body is dietary carbohydrates, mostly from fructose, lactose and starches. The breakdown of these carbohydrates is reliant on enzymes, including, amylases and oligossacharidases; α-glucosidase, lactase and sucrase [25]. The salivary amylase begins breaking down starches in the mouth into maltose, a disaccharide. The next step of carbohydrate digestion occurs in the duodenum where the most available enzymes (pancreatic amylases) continue to break down carbohydrates into disaccharides. The salivary and pancreatic amylases are characterised as endosaccharidases and are only specific for α-1,4 glycosidic bonds [26]. In the duodenum, oligossacharidases cleave the respective oligosaccharides into monosaccharides (mainly glucose) [25]. The glucose becomes available for utilisation to harness energy during metabolism. The glucose, among other monosaccharides resulting from the breakdown by amylases and brush border enzymes in the small intestines, is absorbed through the enterocytes. Selective active transport of D-isomers (mostly D-glucose and D-galactose) occurs through sodium (Na)-coupled secondary active transport symporter known as the Na-glucose transporter 1 (SGLT1) [27]. Active transport ensures a one-directional flow of glucose; that is from the gut to the epithelial cells regardless of the glucose gradient (Figure 1). 

SGLT1 is a high-affinity Na-glucose transporter whose binding is directly influenced by a 2:1 stoichiometric ratio for Na^+^ and D-glucose, respectively [28]. The presence of Na^+^ allows a conformational change in the SGLT1, enabling the D-glucose to bind with higher affinity. Subsequently, the Na^+^ dissociates from its binding site causing a decreased transporter affinity for D-glucose, which is released into the cytoplasm. The SGLT1 completes its cycle in a slow transition to reorient its binding site to the extracellular process [29]. The active movement conferred by Na^+^, K^+^ and ATPases, bringing in D-glucose through SGLT1, creates a steep glucose concentration gradient across the luminal membrane [30]. Afterwards, glucose leaves the cell’s basolateral side by facilitated diffusion using glucose transporters (GLUT2s) to the extracellular medium near blood capillaries [31]. The absorbed glucose within the capillaries is then transported via mesenteric circulation to target cells. 

### 3.2. Regulation of Glucose Metabolism

Blood glucose levels in humans are maintained at a stringent level of approximately 90 mg/dL [32]. Glucose homeostasis is maintained by a balance in the appearance and disappearance of glucose in the bloodstream, regulated by nutritional and hormonal signals. Of note, it is vital to consider processes like carbohydrate digestion and gastric emptying, which directly influence the influx of glucose into the bloodstream. To understand glucose homeostasis, it is also essential to consider and appreciate the contribution of individual tissues involved in glucose metabolism [33]. Under glucose post absorption scenarios, the brain disposes of about 23% of the glucose, about 29% is utilised by splanchnic tissues (liver and gut). The other 25% in glucose disappearance is due to insulin-dependent skeletal tissues, while the rest is used by other tissues such as the heart, adipose tissue and the kidney [34,35].

The major contributors to glucose metabolism in the bloodstream are the pancreatic hormones; glucagon produced by the α-cells, and insulin and amylin produced by the β-cells. The principal roles of glucagon include the breakdown of stored liver glycogen and stimulation of gluconeogenesis and ketogenesis [24]. Thus, glucagon is significantly responsible for glucose appearance. In contrast, insulin and amylin are responsible for postprandial glucose disappearance. Insulin’s roles include promoting glucose uptake by cells and stimulating protein and fat synthesis [36]. Insulin also suppresses postprandial glucagon secretion. The other hormone produced by β cells, amylin, plays a role in suppressing glucagon and slowing down gastric emptying [24]. Moreover, the predominant intestinal *L*-cell produced hormones; glucose-dependent insulinotropic polypeptide (GIP) and glucagon-like peptide-1 (GLP-1), are also responsible for glucose disappearance in the blood capillaries [37,38]. They mediate this by slowing down gastric emptying, suppressing postprandial glucagon secretion, and enhancing glucose-dependent insulin secretion [24].

### 3.3. Glucose Metabolism in Cells

#### 3.3.1. Glucose Uptake

After absorption from the gut, glucose is transported to target cells where its uptake occurs, a process which is insulin-mediated in some cells, including the muscle and adipose tissues [36]. Glucose uptake in the muscles is mediated by glucose transporter 4 (GLUT4), which accounts for approximately 70% of insulin-dependent glucose uptake [39]. Furthermore, intracellular glucose transport into adipocytes, which accounts for about 10% of insulin-mediated glucose, is facilitated by GLUT4. Although glucose uptake by the liver is insulin-independent, it accounts for about 30% of whole-body insulin-mediated glucose disposal. Through intracellular signalling, insulin stimulates glycogen production and inhibits gluconeogenesis and ketone body production [39]. The physiological cellular regulation of glucose uptake is complex; it relies on various factors, including the delivery of glucose to the cells, transport of glucose into cells by glucose transporters like GLUT4 and glycolysis processes, dependent on several enzymatic reactions [40].

#### 3.3.2. Glycolytic Flux

Glycolysis provides some cellular energy and intermediates for more energy production required for metabolism in most organisms, including humans [41]. Moreover, the glycolytic pathway is a source of precursors for biomass production. Over the past century, the mechanisms and regulation of the ten glycolytic steps which yield 2 molecules of pyruvate for every glucose molecule have been well studied. These, together with the lactate dehydrogenase reaction as well as the glucose uptake via transporters and lactate export via monocarboxylate transporters (MCTs), make up several glycolytic flux steps [41]. The hepatic glucose production has been implicated as a significant contributor to blood hyperglycaemia, and its control is dependent on the glycolytic/gluconeogenic flux [32]. There are 10 reactions in glycolysis, and 7 can be used for both glycolysis and gluconeogenesis. The other 3 reactions which include the conversion of glucose-6-phosphate (G6P) to glucose, fructose-1, 6-bisphosphate (F-1, 6-bisP) to fructose-6-phosphate (F6P) and conversion of pyruvate to phosphoenolpyruvate (PEP) are unique to gluconeogenesis [32,42]. The glycolytic metabolite flux in the muscle, adipose tissues and hepatic glucose production is regulated by several vital enzymes which are affected by the relative presence of insulin or glucagon. The regulation and balance of reactions contributing to the glycolytic flux are critical for mammalian physiology as they contribute to glucose homeostasis [43]. Moreover, glucose metabolism dysregulation is a hallmark to diseases, including diabetes and cancer [41]. 

#### 3.3.3. Pathophysiology of Glucose Metabolism

##### Relevance to Diabetes

Dysregulation and poor glucose metabolism lead to the development of diabetes mellitus [44]. Type 1 diabetes mellitus is an autoimmune disease characterised by the destruction of pancreatic β-cells which produce insulin. With the lack of insulin, the body is unable to regulate blood glucose levels. Type 1 diabetes’s pathophysiology also constitutes functional defects in the bone marrow, thymus, β-cells and the immune system as a whole [45]. Type 2 diabetes mellitus (T2DM) occurs when β-cells fail to secrete adequate insulin to keep up with demand, often influencing insulin resistance [46]. Insulin resistance is associated with ectopic fat deposition in the liver and muscles.

Moreover, fat may also amass in the pancreas, which may cause inflammation of the islets of Langerhans, impair β-cell function and eventually lead to their death. T2DM is more prevalent, with over 90% occurrence in people with diabetes, and occurs among adult and older populations, although alarmingly so, youths are demonstrating rising rates [44]. There is a direct relationship between impaired glucose homeostasis and the manifestation of T2DM [47]. Therefore, various processes involved in glucose metabolism, including digestion, absorption of nutrients, glucose uptake by relevant tissues and glycolysis, contribute to hyperglycaemia, especially in people with insulin resistance. Moreover, the control of the enzymes involved with carbohydrate digestion has been studied as a measure to control postprandial hyperglycaemia. The manipulation of glycolytic enzymes and glucose transport have been flagged as potential targets for therapeutic interventions for various ailments, including diabetes and cancer [41]. 

##### Relevance to Cancer

i. Tumour Glucose Metabolism:

Cancerous cells can reprogram glucose metabolism, which is essential for their survival and progression. Tumour progression includes the uncontrolled proliferation of cells with augmented energy production mechanisms to resist metabolic stresses [48]. Cancer cells can switch from oxidative phosphorylation (OXID-P) to glycolysis which yields two pyruvate molecules from the degradation of a single molecule of glucose [49]. The pyruvate is either converted to acetyl coenzyme A (acetyl-CoA), then fully oxidised in the presence of oxygen (normoxic conditions) to produce carbon dioxide and water via the Krebs cycle. However, pyruvate is converted to lactate through an anaerobic glycolytic pathway in the absence or limited oxygen. Most cancer cells rely on the latter process characterised by high glycolysis rates, even when there is oxygen [50,51]. This phenomenon is commonly known as the Warburg effect [52]. The shift of energy metabolism characterised by an elevated glycolytic flux in cancer cells does not reflect defective OXID-P as most tumour cells have normal mitochondrial function [53]. Warburg and colleagues had suggested that the switch in metabolism emanates from mitochondrial damage. However, literature has implicated the metabolic shift to phosphoenolpyruvate conversion to pyruvate catalysed by pyruvate kinase M2, often overexpressed in cancer cells [54]. The pyruvate synthesised through this distinct pathway is converted to lactate by lactate dehydrogenase (LDH) coupled with ATP production, preferably to the formation of acetyl-CoA, which enters the Krebs cycle [55]. Cancer cells increase glucose uptake to adapt to the high energy demands since glycolysis has low energy yields of about 2 ATP molecules per glucose molecule which enters the pathway. This process has been exploited clinically in cancer diagnosis using a radiolabelled glucose analogue [56,57]. The aerobic glycolysis under normoxic conditions has been a target for chemotherapy recently. For instance, inhibition of mitochondrial OXID-P suppressed hepatocellular tumour proliferation [58]. Moreover, inhibition of OXID-P reduced the multidrug resistance of melanoma cells [59]. Therefore, tumour glycolysis, even in normoxic conditions, can be a target for therapeutic efforts. 

ii. Metabolic Phenotypes of Tumour Cells (Hybrid State):

Metabolic phenotypes of cancer cells are the glycolytic phenotype that follows the Warburg glucose metabolism and the non-glycolytic oxidative phenotype [60]. Most cancer cells are of the glycolytic phenotype, and how the metabolic switch occurs in the phenotype is not clear. Glycolytic cancer cells show non-glycolytic character under acidic conditions with an accumulation of lactate. Lactate acidosis is a recurrent result of the Warburg effect in solid tumours [61,62]. The lowered cellular pH inhibits glycolytic enzymes and hence reduces glycolytic flux causing glycolysis suppression. The limited supply of glucose may cause tumour death. Still, under lactate acidosis, the cells switch from the Warburg effect to a non-glycolytic oxidative phenotype which slowly metabolises glucose to ensure cell survival [63]. 

Similarly, in hypoxia or mitochondrial dysfunction, the cancer cells switch from oxidative glucose metabolism to the Warburg metabolism to sustain cell growth [64]. Some cancer cells have been reported to use this metabolic shift to resist or tolerate therapeutic efforts. Breast cancer cells are deemed to resist radiation by shifting from the glycolytic phenotype to OXID-P to produce more ATP necessary for their survival [65]. This behaviour of cancer cells, together with the concept of the Warburg effect, indicates that the idea of a dual metabolic nature or hybrid state is not improbable in stressful cellular conditions [60]. Moreover, the hybrid metabolic phenotype has been reported in aggressive tumour cell lines, including SiHa and HeLa, due to the robust activation of hypoxia-inducible factor-1 (HIF-1) from lactate accumulation [66]. Besides, the hybrid phenotype enables tumours to adapt to oxygen shock when they invade oxygen-rich tissues by switching from the glycolytic phenotype to the non-glycolytic oxidative phenotype [67]. The hybrid Warburg phenotype/OXID-P phenotype enhances tumour cells’ metabolic plasticity supporting cancer invasion, metastasis, and chemotherapy resistance [68]. Hence targeting the hybrid metabolic state may be a plausible therapeutic strategy to eradicate tumour metabolic plasticity [60].

iii. Glycolytic Flux Targets for Therapeutic Efforts:

Glycolysis occurs within the cell cytoplasm and relies on glucose transporters’ (GLUTs) as its substrate importers. In cancer cells, where glycolysis is remarkably high, GLUT1 and Na-glucose linked transporter 1 (SGLT1) are often overexpressed. GLUT3 and GLUT5 are also overexpressed in tumour cells [41]. Inhibitors impeding glucose transport have been reported, including phloretin and ritonavir in combination with metformin, and have entered the clinical trial stage [69,70]. 

**Figure 2:**
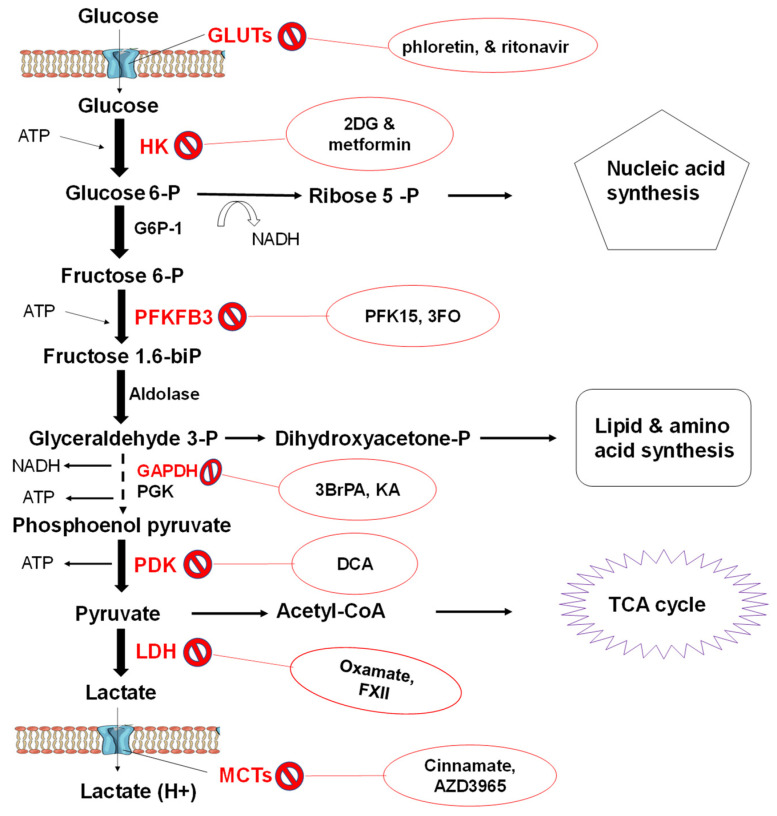
The glycolytic flux process. A summary of the glycolysis pathway with its metabolic intermediates that can be used for various processes by cells. The known glycolytic enzyme inhibitors are presented in red circles and their target enzymes in red. Glucose transporters (GLUTs) and lactate monocarboxylate transporters (MCTs) are shown as transmembrane proteins. Moreover, the enzymes and transporters illustrated may be plausible targets for fucoidan. Adapted from [60].

Upon entry, glucose is phosphorylated to glucose 6 phosphate (G6P) by hexokinases in a rate-limiting step designed to preserve energy in the cell. There are four hexokinase (HK) isoforms with a high affinity for glucose which catalyse this reaction [71]. Among the hexokinases, HK-1 is ubiquitously expressed, whereas HK-2 is expressed in insulin-sensitive muscles and adipose tissue [72]. Moreover, HK-2 is overly expressed in tumour cells [73]. Metformin, an antidiabetic drug, was reported to inhibit the activity of hexokinase partially impairing glucose metabolism and thus suppressing tumour growth in breast cancer [74]. An analogue of glucose, 2-deoxy-D-glucose (2-DG), has been used to inhibit the function of HK by mimicking the glucose substrate [75]. Upon synthesis by HK, glucose-6-phosphate undergoes isomerisation into fructose-6-phosphate with the aid of glucose-6-phosphate isomerase. Fructose-6-phosphate is subsequently phosphorylated to form fructose-1, 6-bisphosphate and fructose-2, 6-bisphosphate, under the effect of phosphofructokinase-1 (PFK1) and PFK2, respectively, using ATP as a phosphoryl donor (Figure 2). 

PFK1 is crucial in the control of glycolysis as high ATP intracellular levels inhibit it. Nevertheless, PFK2 is overexpressed in cancer cells producing excess fructose-2, 6-bisphosphate, which in turn activates PFK1, thus maintaining an elevated glycolytic rate independent of ATP levels [76]. Another isoform of PFKs, namely PFKFB3, which is overexpressed in cancer cells, has been targeted therapeutically using PFK15 (1-(4-pyridinyl)-3-(2-quinolinyl)-2-propen-1-one) [77]. Tanner and colleagues suggested that the enzymes acting on the upper glycolysis mediate control of the glycolytic flux through systematic enzyme overexpression [41]. However, some enzymes in lower glycolysis, including aldolase (ALDOA), glyceraldehyde 3 phosphate dehydrogenase (GAPDH), phosphoglycerate kinase (PGK), pyruvate kinases (PKM1 and PKM2) together with the glycolytic flux enzyme lactate dehydrogenase (LDH) have been reported to have a significant effect on the Warburg process. The enzyme GAPDH has been strategically targeted by several compounds, including koningic acid (KO) and iodoacetate (IO), which are in the preclinical stages [77,78]. PDK also has an inhibitor, dichloroacetate (DCA), which is in the first phase of clinical trials [79], while oxamate is in the preclinical trial phase for regulating LDHA [80] (Figure 2). Finally, one of the essential membrane proteins, monocarboxylate transporters (MCTs), are targeted by cinnamate and AZD3965, which are in the preclinical and phase 1 stages, respectively [81,82].

Although glycolysis yields about 18 times less ATP than mitochondrial oxidation, glycolysis’s accelerated rate achieved 100 times more ATP production than OXID-P [83]. In addition, aerobic glycolysis provides cancer cells with metabolic intermediates; lipids, nucleotides, and amino acids, which are important in the biosynthesis of macromolecules needed during cell proliferation [84]. The glycolytic pathways also produce NADPH and NADH, which act as redox buffers for the cell to avoid the free radical effect of chemotherapeutics [85]. The ability of lactate influx and efflux is vital in the survival of cancer cells through metabolic symbiosis, where the glycolytic and non-glycolytic cells coexist in solid tumours [60]. The lactate is produced and exported by hypoxic glycolytic tumour cells to be imported and utilised by normoxic cells for energy production via mitochondrial OXID-P. This process is mediated by MCTs, where mainly MCT4s are responsible for lactate release and MCT1s for lactate uptake [86]. Recently, in MCF-7 and MDA-MB-231 breast cancer cell lines, there was a correlation in the distribution of MCT isoforms (1 and 4) and expression of LDH isoforms (A and B) [87]. Interestingly, in the MDA-MB-231 cells where LDH was sufficient to produce high titres of lactate from pyruvate, MCT4 was overexpressed.

Conversely, in the MCF-7 cells, there was an overexpression of MCT1 for lactate uptake and LDHB to convert lactate to pyruvate to fuel the TCA cycle [88]. Cancer cells organise their glycolytic phenotypes to achieve maximal energy production for their proliferation. Thus, it is essential to invest research into prospective pharmacological products (such as fucoidan) which may be potential therapeutics that target one or more stages in glucose metabolism pathways unique to cancer cells. 

## 4. Therapeutic Roles of Fucoidan

### 4.1. Significance of Fucoidan as an Anti-Diabetic Agent

There is no doubt that the diabetes burden is increasing yearly and has become the seventh-largest killer disease in the world with its prevalence rising significantly faster in low to medium-income countries than in high-income countries [89]. Diabetes complications include blindness, kidney failure, heart attacks, stroke, and lower limb amputation. Moreover, about 50% of diabetes-related mortality attributed to hyperglycaemia occurs before the age of 70 years [89]. There are currently several remedies available to control T2DM, which is the most common type of diabetes. The leading antidiabetic drug is metformin, a plant derivative, which increases insulin sensitivity, reducing hyperglycaemia [90]. Other commonly used drugs are α-glucosidase inhibitors, including acarbose and miglitol, which slow down starches’ breakdown into glucose [91]. However, these compounds have been associated with side effects, including flatulence, diarrhoea, and abdominal discomfort [92]. Moreover, recently, the FDA recalled metformin with issues surrounding nitrosamine impurity associated with its formulation [93]. These factors, including increasing disease burden, cytotoxicity and low availability of therapeutic drugs necessitates the search for natural, readily abundant remedies like fucoidan. In the past decade, fucoidan’s antidiabetic potential as a novel bio-compound has gained momentum with its therapeutic effects reported at various levels of glucose metabolism (Table 2). Fucoidan has also been implicated in inhibiting dipeptidyl peptidase IV, an enzyme responsible for the rapid degradation of incretin hormones [94] which are known to prevent hyperglycaemia and increase insulin production [95]. Furthermore, fucoidan has been reported to alter gastrointestinal function, including bowel movements. In a study, where type 2 diabetes mellitus (T2DM) patients were enrolled, a high molecular weight fucoidan increased stool frequency and enhanced taste sensitivity, which favoured the management of T2DM [96]. In addition, fucoidan intake increases leptin levels, regulating energy balance by inhibiting hunger, and diminishes fat storage in adipocytes. Notably, there was no associated increase in BMI or blood pressure fluctuations or any other side effects.

The molecular weight and degree of sulphation have been deemed the most important determinants of fucoidan bioactivity, among others [13]. It is challenging to link the structural and chemical composition to function, as individual studies provide inadequate data for a general comparison (Table 2). For example, in Table 2 some studies did not provide molecular weight, or the data was obtained using different methodologies which complicates comparison. However, most fucoidans reported are of medium molecular weight, and the degree of sulphation was above 6%, which could explain their respective bioactivities. Some extracted crude and purified fucoidan samples have been identified as potent inhibitors of the starch digesting enzymes α-amylase and α-glucosidase. Literature has reported that fucoidan extracts isolated from *T. ornate* and *A. nodosum* exhibit an inhibitory effect on the activity of α-amylase [5,14]. In addition, fucoidan from a variety of brown seaweeds, including *F. vesiculosus, E. radiata* and *U. pinnatifida*, have demonstrated significantly higher inhibition potency for α-glucosidase (Table 2) compared to acarbose, which is one of the current therapeutics used to target these enzymes. Therefore, it is vital that more seaweed species are screened, which can target these enzymes and slow down carbohydrate digestion. Slowing down or regulating the activity of these enzymes will directly influence the amount of monosaccharides, especially glucose available for absorption (Figure 1).

It has been established that the absorption of glucose depends on the change in the electrical potential in the small intestinal epithelium. The primary route for the transport of dietary glucose from the intestinal lumen into enterocytes is via the Na^+^/glucose cotransporters, namely SGLT1 and GLUT2 (Figure 1) [28]. Although the role of SGLTs in glucose absorption has been studied extensively, little is known about the regulation of these glucose transport systems. Due to fucoidan properties (including their negative charge), their application in the regulation or manipulation of the SGLTs may prove beneficial for preventing hyperglycaemia leading to glucose pathophysiology. The appearance and disappearance of glucose in the blood are controlled mainly by hormones; insulin and glucagon [24]. Hormonally controlled glucose homeostasis is linked to glucose uptake by insulin-sensitive cells, including muscle cells and adipose cells. The liver is also responsible for hepatic glucose production implicated in hyperglycaemia [32]. Therefore, molecules which may increase insulin sensitivity or directly influence glucose uptake are potential therapeutics. Among other developing remedies, fucoidan extracted from *C. frondosa* has been reported to activate the PI3K/PKB pathway, which regulates insulin production [101]. This fucoidan also triggered the translocation of GLUT4. Another fucoidan from *U. pinnatifida* reduced blood glucose levels and improved insulin sensitivity in mice and decreased basal lipolysis in 3T3-L1 adipocytes which may reduce hyperglycaemia by glucose uptake [102]. Interestingly, *A. molpadioides* was reported to inhibit enzymes involved in glucose metabolism, including hexokinase and pyruvate kinase [104], which are implicated as major players in the glycolytic flux. 

### 4.2. Significance of Fucoidan as an Anticancer Agent

Although there have been considerable advances in medical research in the past years, cancer has remained one of the primary causes of death in the world. Presently, several different treatments such as chemotherapy, radiation therapy, surgery or combinations are used to treat several types of cancers [13]. Chemotherapeutic agents are the primary method of treating various cancers. A range of chemotherapeutic agents, including anthracyclines, methotrexate and folic acid analogues, have been used to treat cancer. These chemotherapeutic agents target rapidly dividing and proliferating cells and commonly deregulated mechanisms within cancer cells [105]. Unfortunately, many of these treatments are indiscriminately toxic and affect normal cells, as well. The toxicity of chemotherapeutics to normal cells limits the amounts that can be used, and therefore, their efficacy [105]. Finding cancer therapeutics that effectively destroy tumours, possess low cytotoxicity and selectively targets the cancerous growth, has focused research towards discovering more tolerable, selective and effective anticancer drugs [105]. Chemotherapeutic cytotoxicity has generated interest in exploiting natural resources such as fucoidan in cancer treatment interventions (Table 3).

Inconsistencies in fucoidan characterisation make comparison linking properties such as molecular weight and sulphate content to biological activity difficult between species (Table 3). However, the importance of molecular weight and degree of sulphation have been established within individual studies. Numerous fucoidans have been shown to elicit anticancer effects against various cancer cell lines. Fucoidan toxicity in tumour cells is reported to occur through various mechanisms, including cell cycle arrest, induction of apoptosis, anti-angiogenesis, inhibition of metastasis and migration or indirectly by activating natural killer cells or macrophages (Table 3) [9]. These anticancer activities observed are likely mediated via multiple signal transduction pathways [9]. The degree of sulphation within fucoidan has been implicated as being crucial for their anticancer activity [17,109].

Nevertheless, fucoidan with no sulphate groups extracted from *S. hornery* demonstrated anti-tumour activity comparable to that of a fucoidan containing a 16.9% sulphate content [116]. This observation may suggest that factors other than the degree of sulphation may be important for fucoidan bioactivity. Fucoidan has the potential to be modified into potent therapeutic compounds against tumour cells as stand-alone agents or in combination with existing chemotherapeutics. The combination of commercial cancer drugs and fucoidan has proven useful as fucoidan showed synergistic effects in improving the potency of the medicines [117]. For instance, an in vivo study in mice demonstrated the synergistic effect of *F. evanescens* fucoidan and the chemotherapeutic drug, cyclophosphamide, in anti-tumour activity against lung adenocarcinoma [118]. Interestingly, in a recent clinical study, LMWF, as a complementary therapy, showed better efficacy than the control fucoidan, which makes fucoidan modification and research essential [9]. Notably, the use of fucoidan for therapeutic purposes shows much potential, however, nothing is used clinically. Therefore, more rigorous research is required to substantiate the clinical benefit of these compounds.

## 5. Potential Double Impact of Fucoidan on Diabetes and Cancer 

**Figure 3:**
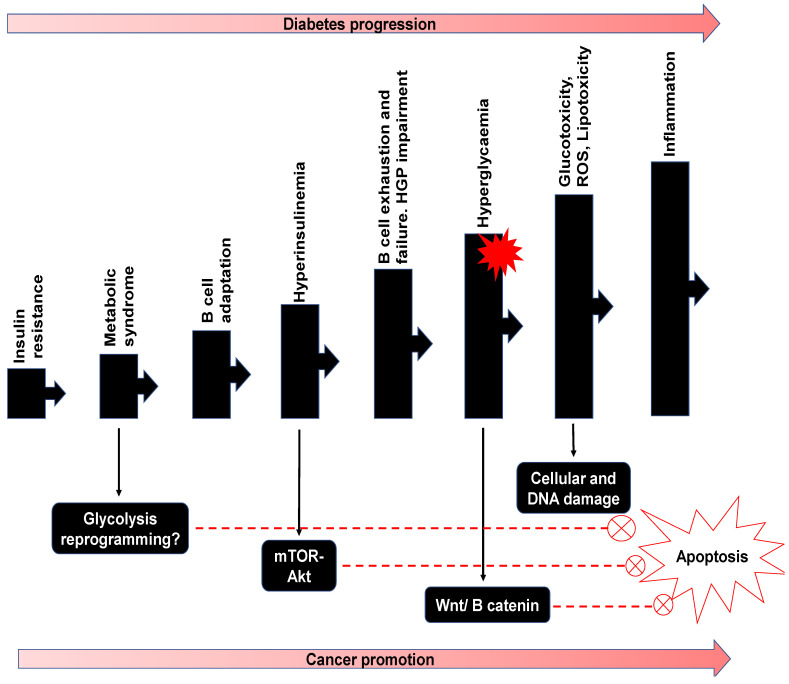
Shared factors in the progression of diabetes and cancer promotion. Insulin resistance, glycolysis reprogramming and β-cell failure, all increase hyperglycaemia. These also alter pathways protecting tumour cells from apoptosis and encourages tumour proliferation. Fucoidan may be useful in regulating one or more pathways which may directly affect glucose metabolism. Adapted from [11].

Epidemiological evidence has shown that individuals with diabetes are at significantly greater risk for multiple types of cancers. Recently, a link between various pathways in diabetes and cancer progression was reported [11]. Tudzarova and colleagues reviewed the clinical association of type 2 diabetes and numerous cancers and highlighted the diabetes-cancer link’s complexity (Figure 3). In general, events leading to T2DM include insulin resistance, pancreatic β-cell impairment, and consequently altered hepatic glucose production (HGP) [119,120]. The resulting hyperglycaemia with the overworked β-cells may lead to metabolic reprogramming and the switch to glycolysis characteristic of cancer development [11]. Hyperinsulinemia is associated with the stimulation of insulin-like growth factor (IGF) and epidermal growth factor (EGF) which activate the mTor-Akt pathway. In addition to hyperglycaemia, which cooperates with the Wnt/β-catenin pathway, inhibition of apoptosis may result in cell damage by glucotoxicity and elevated reactive oxygen species (ROS). Cell responses to ROS cause inflammation which may collaborate with mitogenic and metabolic pathways to initiate or promote cancer progression (Figure 3). Some common disease progression manifestation of diabetes and cancer include hyperinsulinemia, hyperglycaemia, and inflammation [120]. Hence the proposed focus on glucose pathophysiology as the target for the dual control and suppression of diabetes and cancer makes sense. Consequently, fucoidan, in addition to other similar therapeutic compounds, has the potential to be applied in this therapeutic effort.

## 6. Conclusions and Future Perspectives 

Fucoidans from several brown seaweeds have demonstrated a significant role in inhibiting starch digesting enzymes, namely, α-amylase and α-glucosidase, thus, impacting the amount of glucose available for absorption into the bloodstream. However, as far as we are aware, there have been no studies investigating the effect of fucoidan on glucose absorption through SGLTs. Moreover, glucose appearance in the bloodstream is dependent on gastric emptying, which is nutrition- and hormone-regulated. It is not too ambitious to investigate the impact of fucoidan on gastric emptying. Recent studies have established that fucoidan increases insulin sensitivity and lowers postprandial blood glucose, preventing hyperglycaemia in vivo. Furthermore, fucoidan has been implicated in activating pathways which increase insulin production and even glucose transporter molecule translocation. However, very few studies have investigated these aspects, and thus fucoidan could be therapeutically useful. In addition, limited literature is available on fucoidan’s effect on glycolytic enzymes, which are essential in the glucose disappearance from the bloodstream.

In comparison to its reported antidiabetic effects, the anticancer effects of various fucoidans have been intensely studied. Fucoidan has been suggested in playing a role in the regulation of signalling molecules, including, receptor tyrosine kinases and inducing cell cycle arrest and apoptosis [13]. Furthermore, fucoidan suppresses the migration of tumour cells and is deemed to enhance the production of immune cells [121]. However, recent studies have demonstrated the significance of the pathophysiology of glucose metabolism and the glycolytic flux in the development of tumours. Still, little has been done with regards to screening fucoidan. The glycolytic flux and all the steps involved in glucose homeostasis (Figure 1 and Figure 2) may be targets for dual chemotherapeutic efforts against diabetes and cancer. Moreover, fucoidan holds potential as a bioactive compound, and the biodiversity of seaweeds is rich and relatively unexplored. Fucoidan as a marine bioproduct is perceived as less toxic with fewer side-effects, compared to the synthetic chemotherapeutic drugs currently used as antidiabetic and anticancer drugs. However, most fucoidan bioactivity studies involve in vitro and in vivo models in mice. Very few studies have been conducted on human participants; hence cytotoxicity cannot directly be extrapolated from these study designs. Finally, we conclude by stating that fucoidan is a relevant, potentially dual therapeutic agent against diabetes and cancer. Thus, further research on the links between their structures and their bioactivities are necessary.

## Figures and Tables

**Table 1:** A sample of partially characterised fucoidan studied for antidiabetic and anticancer potential.

Source	M_w_ (kDa)	Sulphate Content (*w/w*)	Fucose Content (*w/w*)	Monosaccharide Composition (*w/w*)	Polyphenol Content (*w/w*)	References
*Fucus vesiculosus*	98	15.5 ± 1.1%	94.8%	2.3% xylose, 1.9% galactose	ND	[4,5,19]
*Ascophyllum nodosum*	420	20.6 ± 0.3%	80.1%	14.3% xylose, 5.6% galactose	ND	[3,4,20]
*Sargassum wightii*	637	36 ± 0.60%	53 ± 0.52%	ND	ND	[21]
*Sargassum honeri*	ND	ND	32.5%	23.2% mannose, 27.6% galactose, 4.2% xylose	ND	[4]
*Ecklonia maxima*	470	6.01 ± 0.53%	4.45 ± 0.25%	12.78% fructose, 1.44% galactose, 26.55% glucose, 4.3% mannose, 0.78% xylose	0%	[10,22]
*Turbinaria ornata*	ND	33 ± 0.42%	59 ± 0.69%	ND	ND	[14]
*Undaria pinnatifida*	378	15.02%	39.24%	28.85% xylose, 26.4% galactose, 5.04% mannose, 0.95% glucose	ND	[23]

ND: Not determined.

**Table 2:** The established antidiabetic potential of fucoidan to date.

Fucoidan Source	Harvest Location	MW (kDa)	Sulphate *w/w* (%)	α-Amylase (IC_50_)	α-Glucosidase (IC_50_)	Other	Test System	Reference
*Turbinaria ornata*	India	ND	33 ± 0.42	36.6 μg/mL	ND	Not cytotoxic to normal cells	In vitro	[14]
		ND	ND	250 μg/mL	535.6 μg/mL	Inhibited dipeptidyl peptidase IV (IC_50_ = 55.2 μg/mL)	In vitro	[97]
*Ascophyllum nodosum*	Canada	420	ND	0.12–4.64 mg/mL	13–47 μg/mL		In vitro	[5,20]
*Fucus vesiculosus*	Canada	98	23.7 ± 0.04	No activity	49 μg/mL		In vitro	[5,19,98]
	Russia	735	27	ND	ND	Inhibited dipeptidyl peptidase IV (IC_50_ = 11.1 μg/mL)	In vitro	[94]
*Sargassum wightii*	India	637	36 ± 0.60%	ND	139 μg/mL		In vitro	[21]
		ND	ND	378.3 μg/mL	314.8 μg/mL	Inhibited dipeptidyl peptidase IV (IC_50_ 38.27 μg/mL)	In vitro	[99]
*Cucumaria frondosa*	China	ND	ND	ND	ND	-Activates the PI3K/PKB pathway which regulates insulin production -Activates GLUT4 translocation	In vivo	[100]
Algal extract mixture (*F. vesiculosus* & *A. nodosum*)	Commercial (Italy)	ND	ND	1.49 ± 0.32 μg/mL	0.604 ± 0.004 μg/mL	-Significantly reduced postprandial glucose -Implicated in preventing progression of in non-alcoholic steatohepatitis to T2DM	In vitro & in vivo	[101]
*Undaria pinnatifida*	Commercial (Sigma Aldrich)			ND	ND	-Reduced blood glucose levels and improve insulin sensitivity in mice -Reduction of basal lipolysis in 3T3-L1 adipocytes	In vivo	[102]
	New Zealand	ND	15.02	0.190 ± 0.005 mg/mL	0.137 ± 0.012 mg/mL	-Non-competitive inhibitor of α amylase -Competitive inhibitor of α glucosidase	In vitro	[23,103]
*Acaudina molpadioides*	China	1614.1	26.3 ± 2.7	ND	ND	-Acutely reduced blood glucose levels and improves insulin resistance -Inhibition of glucose metabolism-related enzyme (hexokinase, pyruvate kinase) activities and up-regulation of the PKB/GLUT4 pathway.	In vivo	[104]
*Ecklonia maxima*	South Africa	470	6.01 ± 0.97	No activity	0.29 mg/mL	Mixed inhibitor of α glucosidase	In vitro	[10,22]

ND—Not determined.

**Table 3:** Established anti-tumour activity of fucoidan to date.

Fucoidan Source	Harvest Location	MW (kDa)	Sulphate *w/w* (%)	Cell Line	Mechanism of Action	Test System	Reference
*Bifurcaria bifurcata*	France	ND	ND	NSCLC-N6 cell line	Cell cycle arrest (G1 arrest)	in vitro	[106]
*Saccharina japonica, Undaria pinnatifida*	Japan	ND	0–29%	T-47D and SK-MEL-28 cells	Inhibited cell proliferation and colony formation	in vitro	[107]
NPO organisation fucoidan laboratory	ND	ND	ND	ER+ breast cancer cell line (MCF-7)	Inhibited cell proliferation, induced apoptosis (Caspase 8 activation)	in vitro	[108]
*Cladosiphon okamuranus*	Okinawa Island	ND	Native 13.5% and over-sulphated 32.8%	Human leukaemia cell line (U937 cell)	Native; very weak anti-proliferative activity Over sulphated; Induced apoptosis via caspase-3 and -7 activation-dependent pathways	in vitro	[109]
*Fucus vesiculosus* (Sigma)	ND	ND	ND	HT29 colon cancer cells	Induced G1 cell cycle arrest (induced p21WAF1 expression; suppressed cyclin and cyclin-dependent kinase expression); induction of apoptosis and angiogenesis	in vitro & in vivo	[110]
Fucoidan (Sigma)	ND	ND	ND	Human bladder carcinoma cell lines (5637 and T-24)	Cell growth inhibition via p21WAF1-mediated G1-phase cell-cycle arrest by activation of AKT	in vitro	[111]
*Undaria pinnatifida*	ND	ND	ND	Human lung cancer A549 cells	Apoptosis induction (activation of ERK1/2 MAPK pathways; downregulation of p38, PI3K/Akt signalling)	in vitro	[112]
*Sargassum hemiphyllum* (Hi-Q Marine International Ltd.)	ND	ND	ND	Human hepatocellular carcinoma cells	Inhibits angiogenesis and metastasis of tumour cells (regulation of miR-29b-DNMT3B-MTSS1; inhibition of TGF-β receptor and Smad signalling)	in vitro	[113]
*S. hemiphyllum*, (Hi-Q Marine International Ltd.)	ND	LMWF	ND	Hypoxic human bladder cancer cells (T24) cells; Female athymic nude mice (BALB/c)	Inhibits angiogenesis and tumour growth (inhibition of HIF-1/VEGF-regulated signalling pathway)	in vitro & in vivo	[114]
*F. vesiculosus* (Sigma) *Sargassum sp*	ND	ND	34.2% 38.4%	Lewis Lung Carcinoma cells (LCC); Melanoma B16 cells	Induced apoptosis by fragmentation and condensation of chromatin	in vitro	[115]

ND: Not determined.

## Data Availability

Not applicable.

## Abbreviations

2-DG	2-Deoxy-D-glucose
Acetyl-CoA	Acetyl coenzyme A
DCA	Dichloroacetate
F-1, 6-bisP	Fructose 1.6 phosphate
F-6-P	Fructose 6 phosphate
GAPDH	Glyceraldehyde-3-phosphate dehydrogenase
GLP-1	Glucagon-like peptide-1
G-6-P	Glucose 6 phosphates
GLUT	Glucose transporter
GIP	Glucose-dependent insulinotropic polypeptide
HGP	Hepatic glucose production
HK	Hexokinases
HMWF	High molecular weight fucoidan
IGF	Insulin-like growth factor
IO	Iodoacetate
KO	Koningic acid
LDH	Lactate dehydrogenase
LMWF	Low molecular weight fucoidan
MCTs	Monocarboxylate transporters
Na^+^	Sodium ion
ROS	Reactive oxygen species
SGLT	Na-glucose transporter
OXID-P	Oxidative phosphorylation
PFK1	Phosphofructokinase
T2DM	Type 2 diabetes mellitus
